# Promotion of breast cancer by β-Hexachlorocyclohexane in MCF10AT1 cells and MMTV-neu mice

**DOI:** 10.1186/1471-2407-7-130

**Published:** 2007-07-17

**Authors:** Patrick S Wong, Fumio Matsumura

**Affiliations:** 1 Center for Health and the Environment-John Muir Institute of the Environment, Department of Environmental Toxicology. University of California, Davis, CA 95616, USA

## Abstract

**Background:**

Exposure to β-Hexachlorocyclohexane (β-HCH), a contaminant of the hexachlorohexane pesticide lindane, has been implicated as a risk factor in the development of breast cancers in epidemiological studies. Previous studies in our laboratory have demonstrated the ability of β-HCH to elicit its actions via a ligand-independent activation of the estrogen receptor through increased c-Neu (= erbB_2 _or HER-2) expression and kinase activation in both the BG-1 and MCF-7 cell lines. In addition, long term exposure (33 passages) to β-HCH was shown to promote the selection of MCF-7 cells which exhibit a more metastatic phenotype.

**Methods:**

In this current study, we decided to investigate the long-term effects of β-HCH in both the MCF10AT1 cell line which was derived from a normal epithelial cell line by stably transfecting a mutated c-Ha-ras and a MMTV-Neu mouse model for mammary cancer *in vivo*. MCF10AT1 cells were exposed for 20 passages with β-HCH, 4-OH-Tamoxifen (Tam), or 17-β-estradiol (E_2_) after which cells were analyzed for proliferation rates and mRNA expression by RT-PCR. In our *in vivo *studies, MMTV-Neu mice were injected with β-HCH and observed for tumor formation over a 70 week period.

**Results:**

β-HCH and Tam selected MCF10AT1 cells demonstrated increased mRNA expression of MMP-13 (collagenase-3) a marker of increased invasiveness. β-HCH treatment was also seen to increase the expression in a number of proto-oncogenes (c-Neu, Cyclin D1, p27), cell status markers (Met-1, CK19), and the inflammatory marker NFκB. Previous studies, have demonstrated the role of these markers as evidence of malignant transformations, and further illustrate the ability of β-HCH to be carcinogenic. To demonstrate β-HCH's tumorigenic properties in an *in vivo *system, we used an MMTV-Neu mouse model.

MMTV-Neu is a c-Neu overexpressing strain which has been shown to spontaneously develop mammary tumors at later stages of aging. In this experiment, β-HCH exposure was shown to both accelerate the appearance (~8 weeks for median tumor-free period) and incidence (~25% increase at the end of the test period) of tumors when compared to control mice receiving only the corn-oil vehicle.

**Conclusion:**

Based upon these results, it was concluded that β-HCH does act as a breast cancer promoter which exerts its tumorigenic activity via increased c-Neu expression.

## Background

It is well known that the etiology of human breast cancer is significantly affected by environmental factors. One of those factors frequently discussed as potential cancer promoters is exposure to a class of pollutants known as organochlorine compounds. These compounds include chlorinated pesticides such as DDT, hexachlorcyclohexane (HCH), chlordane, and industrial chemicals such as PCBs and dioxins. Many members of this class of chemical pollutants have been shown to act as both as estrogenic compounds and as tyrosine kinase stimulators. β-Hexachlorocyclohexane (β-HCH), which is the focus on our current study, is a contaminant of the pesticide γ-hexachlorocyclohexane (γ-HCH) or lindane. In previous studies, β-HCH has been shown to activate c-Neu (also known as erbB_2 _or HER2) kinase in breast epithelial cells [1] and ovarian cancer cells [2]. The high lipophilicity of β-HCH, together with its extreme persistence (i.e. the estimated half life of 10 years in human tissues) due to its resistance to metabolism or to other environmental cleansing forces, makes it extremely persistent and bioaccumulating in fatty tissues such as breast tissues. A Finnish epidemiological study has revealed that the average concentration of β-HCH in the breast tissues of cancer patients is 130 μg/kg fat, compared to 80 μg/kg fat for controls (equivalent of approximately 130 and 80 ppb, respectively). It must be noted that based on this study, the difference of a mere 50 ppb could affect the carcinogenic outcome if we accept their view. The authors of the study concluded that this organochlorine compound is a significant risk factor in the development of breast cancers [3].

While there are still questions regarding the meaning of such epidemiological correlations or the existence of the correlation itself, most of the experts would agree that lifetime exposure to strong estrogenic compounds could increase the risk of developing breast cancer. Such an opinion is a reasonable one since total lifetime exposure to estrogens is one of the primary determinants in the development of this type of hormone sensitive cancer, and consequently many organochlorine pesticides, among a host of other xenobiotics, have been shown to act as estrogenic agents.

Curiously, however, many of the environmental estrogens identified thus far have been to be mostly weak estrogen receptor (ER) agonists. Our lead compound, β-HCH, also belongs to the same type of xenoestrogen which does not show any binding affinity to ER [4,5] but acts in an estrogen mimicking manner though its action to stimulate c-Neu kinase [1,6]. β-HCH was also found to be a positive xenoestrogen in a fish (Medaka) assay [7] as well.

In this study, β-HCH has been chosen as a prototype of this class of environmental pollutants for the purpose of testing our hypothesis that they act as breast cancer promoters though their action to stimulate c-Neu kinase activity. This hypothesis has been based on our previous observation that β-HCH activates tyrosine kinase associated with c-Neu *in vitro *in MCF-7 cells [1,6]. However, whether such an action of β-HCH results in the development of highly transformed breast cancer under chronic exposure conditions both *in vitro *and *in vivo *have not been established yet. The main objectives of this investigation were: first, to characterize the specific feature of breast epithelial cells selected and promoted through chronic exposure to β-HCH and second, to demonstrate that such exposure to this compound indeed results in the development of breast cancer *in vivo *in an experimental animal model. Such a mode of promotion is expected to be most effective in cells with pre-existing genetic risk factors such as c-Neu overexpression. Because of this fact, we have selected Ha-ras and c-Neu expressing MCF10AT1 cells *in vitro *and MMTV-neu mice for our *in vivo *model to conduct our studies in order to gain insights to the basic mechanism of breast cancer promotion through c-Neu kinase activation.

## Methods

### Materials

17-β-Estradiol (E_2_) and 4-OH-Tamoxifen (Tam) were purchased from Sigma Biochemicals (St. Louis, MO). β-hexachlorocyclohexane (β-HCH) was purchased from Chem Service (Westchester, PA). E_2 _and pesticides were kept as high concentration stock solutions in ethanol. Human recombinant EGF other media supplies were obtained from Gibco BRL (Gaithersburg, MD).

### Cell culture

#### Source and Maintenance

MCF10AT1 cells were obtained from the Michigan Cancer Foundation and maintained in phenol red free Dulbecco's modified Eagle's medium with F12 nutrient mixtures (DMEM/F12; Gibco, BRL) and supplemented with 2.5% heat-treated equine serum, 20 ng/mL EGF, 100 U/mL penicillin, and 100 μg/mL streptomycin at 37°C under 5% CO_2 _conditions.

#### Selection Process

For each subline, cells were grown in culture media containing either E_2 _(1 nM); Tam (1 nM), β-HCH (1 μM), or just the 0.1% ethanol vehicle (control). Media and chemicals were refreshed twice a week and the plates were reseeded when plates reached 80–90 percent confluency (on average every 10–14 days). For each treatment, three separate plates were prepared and passaged independently. Cell passaging was accomplished by trypsinzation and reseeding of 1 × 10^6 ^cells (approximately 2.5% of a near confluent plate) into a 100 mm plate containing the above media and chemicals. For the quantification of our long-term studies, each reseeding event constituted one passage.

### Fixation and staining of selected MCF10AT1 cells

In a six-well plate, 1,000,000 cells from the selected sublines were seeded (without selection chemicals). After reaching confluence (normally 3–4 days), media was refreshed twice (again without selection chemicals) for an additional week. Afterwards, cells were fixed by 10% Formalin (in PBS) for 30 minutes and stained with Giemsa for 1 minute. After which plates were washed of extra dye solution with tap water repeatedly. Cells were the observed under a compound microscope for morphology.

### Proliferation test assay

After 20 passages in our test chemicals, cells were reseeded for cell proliferation assays. Cells were trypsinized from a starter plate and seeded at a concentration of approximately 100,000 cells per well in a 12 well plate (Hemocytometer counting) or 10,000 per well in a 96 well plate (Promega-AQueous one cell proliferation assay). In most cases, 2 samples from an independent triplicate of a similarly treated selected cell line were chosen (i.e. N = 6). It must be noted that none of the selection chemicals (i.e. E_2_, Tam or β-HCH) were added to the cell culture media used during this assay. At 24, 48 and 72 hours after plating, cells were trypsinized (hemocytometer counting) or analyzed directly for MTS reduction (AQueous assay). Trypan blue exclusion (0.08% dye) was used to determine viable cells (normally >96% of total cells).

### mRNA analysis of gene expression

Cells that have been selected for 20 passages in our test chemicals were trypsinized and reseeded (500,000) into 60 mm dishes in our culture media. Note: no additional selection chemicals (i.e. E_2_, Tamoxifen, β-HCH) were added to the media during this analysis. After 24 hours, the media was refreshed. After an additional 24 hours, mRNA was extracted from the cells using the Qiagen (Valencia, CA) RNAeasy kit. cDNA was prepared from the extracted mRNA using the Omniscript Reverse Transcriptase Kit (Qiagen) and analyzed by real-time PCR on a Roche lightcycler.

Real-time PCR was performed on a Roche Molecular biochemical "Light Cycler". Samples were prepared using the Light cycler DNA Master SYBR green I kit. After sample preparation, cDNA was analyzed with the following primer pairs:

c-Neu :   5'-AGC TGG TGA CAC AGC TTA-3' FP [8]
          5'-TGG TTG GGA CTC TTG AC-3' RP

ERα       5' CAA GCC CGC TCA TGA TCA A-3' FP
(228 bp)  5'-CTG ATC ATG GAG GGT CAA ATC CAC-3' RP

Cyclin D1 5' GGA TGC TGG AGG TCT GCG A-3' FP
(146 bp)  5'-AGA GGC CAC GAA CAT GCA AG-3' RP

p21       5'-ATT AGC AGC GGA ACA AAG AGT CAG ACA T-3' FP
(318 bp)  5'-CTG TGA AAG ACA CAG AAC AGT ACA GGG T-3' RP

p27       5'-AAC GTG CGA GTG TCT AAC GG-3' FP
(164 bp)  5'-CTT CCA TGT CTC TGC AGT GC-3' RP

VEGF      5'-AAG GAG GAG GGC AGA ATC AT-3' FP
(226 bp)  5'-ATC TGC ATG GTG ATG TTG GA-3' RP

IGF-IR    5'-ACA GAG AAC CCC AAG ACT GAG G-3' FP
(285 bp)  5'-TGA TGT TGT AGG TGT CTG CGG C-3' RP

c-met     5'-CAG GCA GTG CAG CAT GTA GT-3' FP
(201 bp)  5'-GAT GAT TCC CTC GGT CAG AA-3' RP

CK19      5'-CTA CAG CCA CTA CTA CAC GAC-3' FP
(148 bp)  5'-CAG AGC CTG TTC CGT CTC AAA-3' RP

MMP-12    5'-ACA CAT TTC GCC TCT CTG CT-3' FP
(192 bp)  5'-CCT TCA GCC AGA AGA ACC TG-3' RP

MMP-13    5'-AAC ATC CAA AAA CGC CAG AC-3' FP
(166 bp)  5'-GGA AGT TCT GGC CAA AAT GA-3' RP

NFκB      5'-CAC TTA TGG ACA ACT ATG AGG TCT CTG G-3' FP
(406 bp)  5'-CTG TCT TGT GGA CAA CGC AGT GGA ATT TTA GG-3' RP

β-Actin   5'-GGA CTT CGA GCA AGA GAT GG-3' FP
(234 bp)  5'-AGCACT GTG TTG GCT TAC AG-3' RP

The PCR cycling conditions were 95°C for 15 s, 60°C for 20 seconds and 72°C for 10 seconds

### MMTV mice treatments

MMTV-neu mice were obtained from Jackson Laboratories (West Sacramento, CA) and separated into control and β-HCH treated groups. Each group contained 37–39 virgin female mice. Mice were injected with 33 mg/kg β-HCH (in corn oil:acetone; 90:10 mixture) or just the vehicle solution at 6, 10, 14 and 18 weeks. Mice were housed at normal temperature, food, and water conditions and examined twice weekly for tumor formation in the mammary regions. First palpable tumor time was used to calculate tumor latency in animals.

### Statistical Analysis

#### For proliferation and mRNA expression experiments

Several independently prepared batches of cells for each treatment group were assessed simultaneously to obtain means and standard deviations among different batches (i.e. "N" values shown in figure captions). ANOVA and Fisher's least-significant differences test (SYSTAT 11 Computer Software) were used to determine the significance of difference between control and chemically treated groups. A probability value of less than 0.05 was regarded as significant. Error bars in the figures indicate standard deviations from the mean.

#### For MMTV mouse tumor studies

The tumor free interval for mice in the β-HCH and corn-oil only treat groups were compared using the Cox proportional hazard model and the log rank test for comparison of significance between survival curves (MedCalc computer software).

## Results

### Observations on phenotypic changes in MCF10AT1 cells exposed to chemicals for 20 passages

#### Cellular morphology in a confluent system

**Figure 1:**
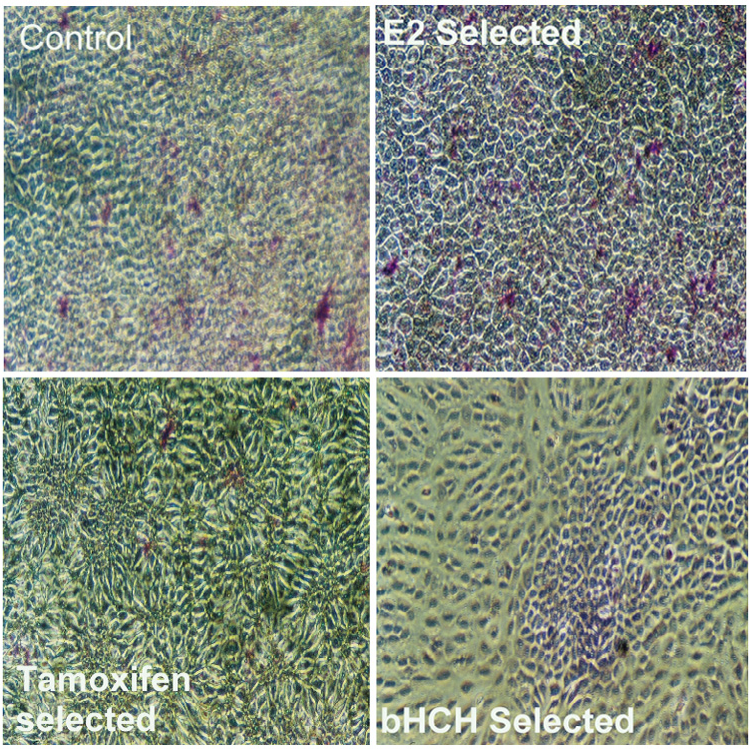
Giemsa stained MCF10AT1 sublines (Control, E_2_, 4-OH-Tamoxifen and β-HCH selected) grown for one week after reaching confluency.

After exposure for 20 passages with our test chemicals, cells were plated and grown to confluence in six-well plates. After cells reached confluence, media was refreshed twice for an additional week. Cells were then fixed, stained with Giemsa and observed under a microscope (Figure 1). There were no observable differences between independent, yet similarly treated sublines. In regards to comparisons between sublines receiving different chemical treatments, both the control and E_2 _selected MCF10AT1 cells (Fig 1A and 1B, respectively) exhibit similar morphology with relatively consistent cellular densities and a lack of secondary formations. E_2 _selected cells, however, did show a more densely packed "cobblestones" appearance with the presence of islands consisting of well packed larger cells. Tam selected MCF10AT1 cells (Fig 1C), exhibited spindle shaped cells with clear foci formation indicating additional cellular transformation of the MCF10AT1 cell line consistent with the loss of contact inhibition, allowing the cell's ability for growth beyond confluency. On the other hand, β-HCH selected MCF10AT1 cells (Fig 1D) showed two types of mixtures of cells without distinct forming foci. One type of cells formed large areas of stationary cobblestone assemblies similar to E_2 _treated cells showing a degree of varying cell sizes of cell densities with large nucleies. The second type of cells showed well dispersed (i.e. less well packed than the first type), spindle shaped cells which did not form either foci or islands of packed assemblies of cells. This observation indicates that while β-HCH exposure also appears to promote and select specific phenotypes in the MCF10AT1 cell line, its actions are disparate from that which occurs in Tam treated cells since there is no visible foci formation.

#### Changes in baseline cellular proliferation

**Figure 2:**
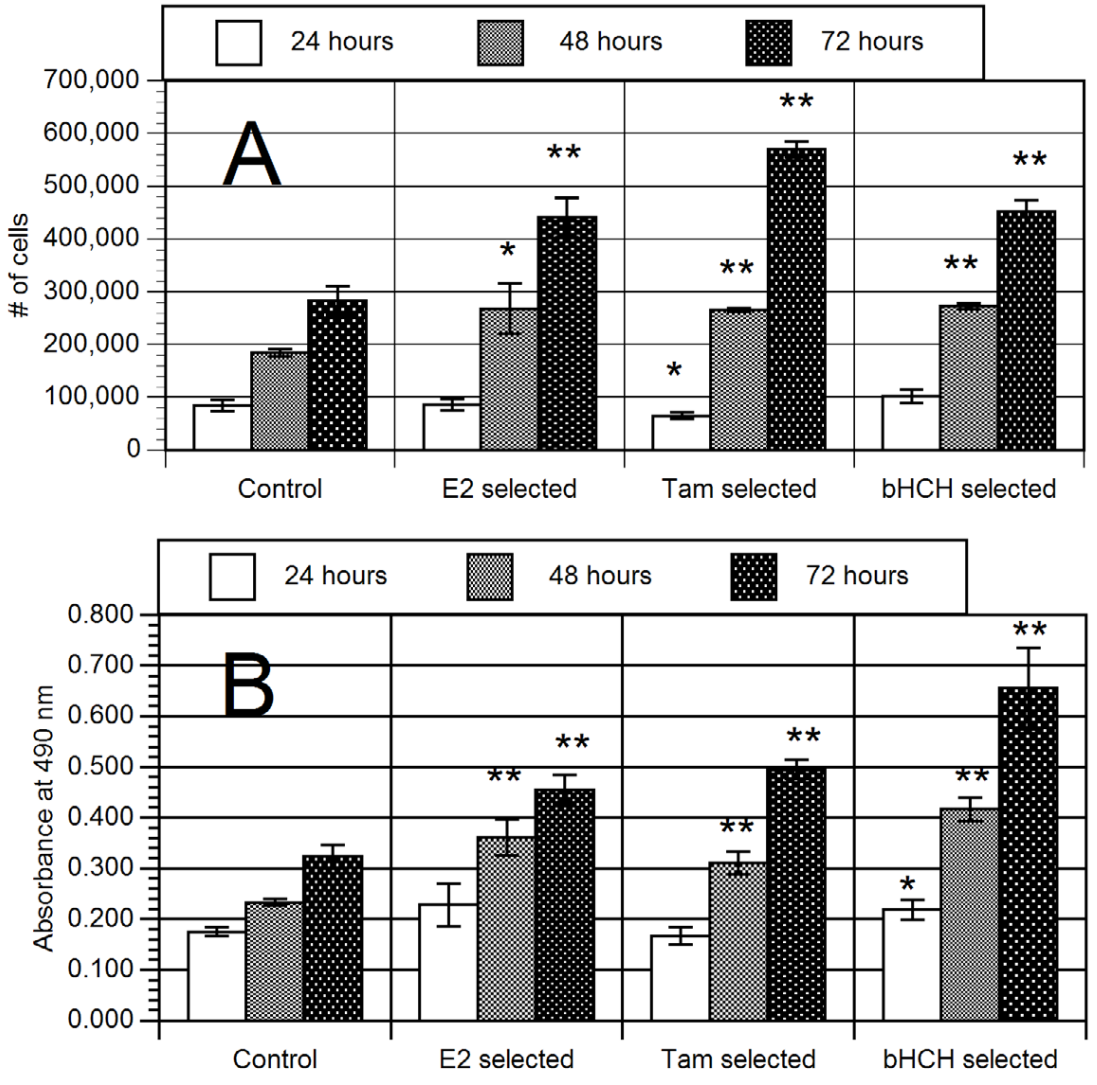
Measurement of cell proliferation in MCF10AT1 sublines (Control, E_2_, Tam and β-HCH selected) as determined by (A) cell counts or (B) MTS bioreduction at 24, 48 and 72 hours after initial cell plating. N = 5–6 for both experiments *p < 0.05, ** p < 0.01 versus control.

After exposure for 20 passages with our test chemicals, cells were reseeded and grown in culture media without the influence of selection chemicals. Cells were then quantitated via hemocytometer counting (Fig 2A) or MTS reduction (Fig. 2B). MTS reduction to its formazen product, has been established as a directly proportional to amount of living cells [9]. At 24 hours, there was, in general, no significant differences in cell number amount the cell groups indicating similar plating efficiencies. Over the next two successive 24 periods, all chemically selected subgroups of long-term selected lines demonstrated increased cell proliferation when compared to the control group. In particular, Tam and β-HCH treated cell lines had nearly double the increase in cells over successive 24 hour periods when compared to the control group. Interestingly, the difference between the two assay methods with regards to proliferation (i.e. C < E_2 _= β-HCH < Tam for 2A but C < E_2 _= Tam < β-HCH for 2B) may be related to an observation during hemocytometer analysis. It was noted that β-HCH and E_2 _cells appeared to be slightly larger than both the Tam and control vehicle treated cells which may account for the more proportional increase in MTS reduction activity.

#### Changes in mRNA expression (Table 1)

**Table 1:** Molecular Profiles of MCF10AT1 sublines (Control, E_2_, Tam and β-HCH selected). The markers shown based on mRNA expression of genes determined though quantitative RT-PCR. Values represent fold-increase of a marker's expression versus β-actin of a selected subline when compared to control (ethanol vehicle). N = 5–6 for each experiment * p < 0.05 and **p < 0.01 vs. treatment versus control.

**MCF10AT1 sublines selected for 20 passages in DMEM/F12 media containing:**				
	Control	E2 (1 nM)	Tam (1 nM)	β-HCH (1 uM)

**Proto-oncogenes**				
cNeu	1.00	1.64*	1.79*	2.73**
Cyclin D1	1.00	1.84*	1.88*	2.50**
p21	1.00	2.05*	1.07	1.19
p27	1.00	2.02*	1.73*	2.80*
**Cell Status**				
VEGF	1.00	3.12*	0.72	1.34
IGF-IR	1.00	1.99*	2.79*	3.77**
c-met	1.00	2.06**	0.66*	1.26
CK19	1.00	0.78*	0.72*	1.67*
**Invasive**				
MMP-12	1.00	0.85	0.41*	3.50*
MMP-13	1.00	0.86	18.51**	14.12**
**Inflammation**				
NFkB	1.00	3.20**	0.24*	2.59**

After exposure for 20 passages with our test chemicals, cells were reseeded and grown in standard culture media without the influence of selection chemicals. The mRNA from these cells were then extracted and quantitated for expression using real-time RT-PCR. Expression of various genes were normalized with respects to β-actin for each sample. These normalized values were then expressed in Table 1 relative to the expression of the same marker in the control group. Molecular profiles were constructed based on the selected cell line's ability to express various markers of transformation (c-Neu, cyclinD1, p21, p27(waf)), cell status (VEGF, IGF-R, c-Met, CK19), invasiveness (MMPs -12 and -13) and inflammation (NFκB). In general, all sublines exhibited increase markers of transformation. Curiously, both cell status and inflammatory markers were upregulated in β-HCH treated cell lines, down-regulated in the Tam treated cell lines and demonstrated mixed results in E_2 _selected cell lines. Among the expression of the invasive MMP markers, MMP-13 was very high in both Tam and β-HCH selected cells and MMP-12 was elevated only in β-HCH selected cells. These results indicate that while all three of our chemicals were able to promote specific phenotypes in the MCF10AT1 cells lines, each of the selected lines show distinct characteristics, implying that the nature of these transformations may be occurring via differing mechanisms.

### *In vivo *exposure of β-HCH in MMTV-neu mice

#### Tumor Formation

**Figure 3:**
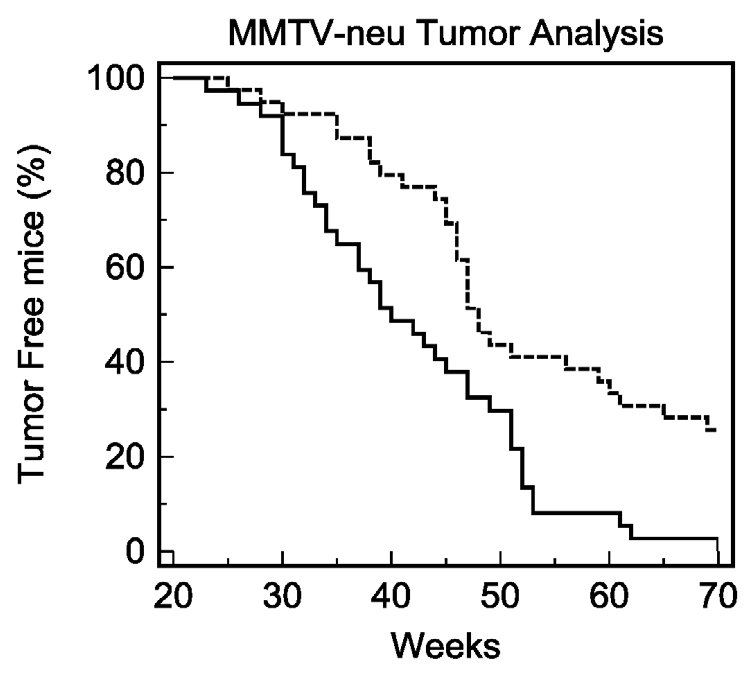
Kaplan-Meier plot of tumor formation in c-Neu MMTV mice injected with β-HCH (solid line) or corn-oil vehicle (dashed line). P = 0.0004 for significance between curves as determined by a comparison of survival curves (log rank test). N = 37 (β-HCH treated group) and 39 (control group)

Previous *in vitro *cellular studies in our laboratory using MCF-7 cells [10] characterized those selected through 33 passages of exposure to β-HCH be ER positive, c-Neu over-expressed, rapidly proliferative and invasive. Previous studies from our laboratory have consistently shown that the acute primary action of β-HCH in MCF-7 cells is rapid activation of c-Neu tyrosine kinase [1,6,11]. Accordingly we have formulated a hypothesis that persistent c-Neu kinase activation for long time periods lead to promotion of malignant phenotype of affected cells. To test this hypothesis *in vivo *we decided to utilize a MMTV-Neu mouse model for mammary cancer, according to the protocol of Yu *et al*. [12]. This transgenic strain is an ideal model for our studies due to its overepression of the c-Neu receptor and its innate ability to spontaneously form mammary tumors. Six-week old virgin female mice were injected with β-HCH (33 mg/kg) or the corn oil vehicle. Injections were repeated 3 additional times on a monthly basis. Mice were inspected weekly for tumor formation by palpations and a Kaplan-Meier survival curve on tumor occurrence or lack thereof was constructed. (Figure 3). The results showed that β-HCH exposure accelerates the appearance of breast tumors in these mice to the extent that the median survival time point of tumor formation in 50% of the population was eight weeks earlier in the β-HCH treated group than control (40 weeks vs 48 weeks). Furthermore, β-HCH treatment caused 100% tumorgenesis while vehicle treated control groups showed only 75% tumorgenesis even at the end of 70 weeks test period.

## Discussion

While previous studies in MCF-7 cells have established that long-term exposure to β-HCH, Tam [10] and E_2 _resulted in increases in metastatic potential via increased invasiveness and expression of metastatic markers, we did not get the overall perspectives of how applicable that finding is to other types of breast cancer cells or how unique is β-HCH in comparison to other cancer promoters in causing this type of transformation. Based on background information obtained from these previous studies, we decided to compare the ability of all three of these compounds to promote changes in the metastatic potential in the epithelial MCF10AT1 cell line. The MCF10AT1 cell line was chosen based on its known background of Ha-ras mutation as the genetic risk factor introduced to the non-transformed MCF10A line, and its ability to be transformed into a variety of a carcinomas when they were xenographed into the breast fat pad of nude mice [13,14] as well as its well defined expression of both the estrogen and c-Neu receptors. When this cell line was subjected to β-HCH, E_2_, or Tam exposure for 20 passages, we could clearly recognize that they all express more transformed phenotypes judging by the changes in their cellular morphology, and proliferation rates. The wide difference among those selected sublines in terms of their cell morphological shape and density of the transformed MCF10AT1 cells indicated that there may be other fundamental differences among them. This led us to examine their molecular profiles. When we examined their molecular profiles through the use of a quantitative RT-PCR approach, it became also apparent that there are distinct differences among all 3 selected lines and control (Table 1) although all 3 selected cell lines showed some common markers of transformation such as elevated mRNA expression of cyclinD_1_, and p27 (kip) genes. For instance, the increase in mRNA for c-Neu gene was more significant upregulated in β-HCH group when compared to the Tam and E_2 _treated group. Interestingly, significant up-regulation of VEGF and p21(waf) mRNA expression could be observed only in E_2 _selected line. These observations indicated that there are clear signs of differences in the patterns of mRNA expressions among all three selected sublines. With respect to the markers for the transformed cell status, VEGF induction was highest in E_2_-selected line, but that of CK19 (cytokeratin) was most significant in β-HCH-selected ones. In contrast, the Tam selected line showed reduced expressions in all status markers. A significant finding on the markers of invasiveness is that for MMP-13 (collegenase 3) of which mRNA expressions were tremendously upregulated from control (selected with the vehicle only) in both β-HCH and Tam selected lines but not at all in E_2 _selected one. Since MMP-13 (collagenase-3) expression is a well-accepted marker of malignancy of mammary epithelial cells [15], we have decided that in future studies we should rely on this mRNA expression as one of the markers in judging the extent of malignant transformation. By contrast, up-regulation of NF-κB, a marker of inflammation was up-regulated in E_2 _and β-HCH selected cells but not in Tam selected ones. Another characteristic seen only in β-HCH selected cells is the increased mRNA expression of IRS-1, insulin receptor substrate protein. Interim conclusions we have derived from this set of profiling tests are: (a) both β-HCH cells demonstrated the most significant elevated c-Neu mRNA expression, (b) judging by those invasiveness markers, particularly MMP-13, β-HCH and tamoxifen selected cells are more transformed and (c) both β-HCH and E_2 _selected cells show the sign of inflammation, while Tam selected line is definitely does not. One key observation is that despite these signs of significant transformation of β-HCH selected cells, they still maintain high levels of expression of cytokeratin (CK19), which is a marker of differentiated non-transformed mammary epithelial cells.

The differing molecular profiles in the RT-PCR results illustrate that while all cells appear to be transformed (with respect to protoncogene upregulation), albeit at varying degrees, there are possible common mechanism in which may lead to similar phenotypical changes. For example, proliferation rates appear to be related to increased invasiveness which where seen in both the Tam and β-HCH selected cells but to a lesser extent in the E_2 _selected cells. In the case of E_2 _and β-HCH selected sublines, the increase in their proliferative activities do not appear to correlate with foci-like formation. Yet, these two sublines do show increased expression of inflammatory marker, NFκB, which is accompanied with up-regulation of VEGF. It has been shown that activation of NFκB is positively linked to a subset of estrogen receptor-positive human breast cancer, which represents clinically aggressive ER-positive breast cancer. Thus, while much more work would be needed to firmly establish the exact molecular phenotype of β-HCH selected cells, the current study results have established that β-HCH is a definite tumor promoter *in vivo*, and that the molecular profile of β-HCH selected cells is distinct as compared to those MCF10AT1 cells selected by E_2 _or Tam.

While there has been a number of ecotoxicological and epidemiological studies linking organochlorine exposure to increased incidences of breast cancers, this study is the first one to demonstrate the direct effect of organochlorine exposure to increased tumor formation in an *in vivo *system (Figure 3). It must be emphasized that the selection of this MMTV-neu model was based on the special characteristics of β-HCH exposed epithelial cells to show high mRNA expression activities of c-Neu and its kinase. We reasoned that in mice already constitutively expressing c-Neu, which is particularly selectively concentrated in mammary tissues, the action of β-HCH would likely be manifested clearly. Results illustrating the ability of β-HCH to promote earlier onset time and to increase the percentage of tumorgenicity in MMTV-neu mice indicate that this organochlorine is a powerful cancer promoter *in vivo*. The β-HCH treated mice had an onset time of nearly three months earlier than corn-oil treated mice for 25% tumor incidence (32 weeks vs 44 weeks). In addition, nearly a quarter of control treated MMTV-neu mice did not have any tumor formation even after 70 weeks. This set of data illustrates that c-Neu overexpression in mammary epithelial cells is a definite risk factor, and that β-HCH is a definite tumor promoting agent *in vivo*, at least in this mouse breast cancer model.

## Conclusion

Based on our results which indicate that β-HCH exposure increases the metastatic characteristics in the MCF10AT1 cell model and tumor formation in the MMTV-neu mouse model, we conclude that β-HCH can act as a breast cancer promoter via its ability to promote c-Neu overexpression.

## Abbreviations Used

ER, estrogen receptor; E_2_, 17-β-Estradiol; Tam, 4-OH-Tamoxifen; EGF, epidermal growth factor; HCH, hexachlorocyclohexane; MMTV, Mouse mammary tumor virus; MTS, 3-(4,5-dimethylthiazol-2-yl)-5-(3-carboxymethoxyphenyl)-2-(4-sulfophenyl)-2H-tetrazolium

## Competing interests

The author(s) declare that they have no competing interests.

## Authors' contributions

This study was conceived jointly by both PSW and FM. PSW was responsible for experimental design and completion of all laboratory work contained in this article. FM also participated in the design and coordination of the work involved. The manuscript was drafted by PW. Both authors approve and read the final manuscript

## Pre-publication history

The pre-publication history for this paper can be accessed here:


